# Supplementary material for: Chromatographic Determination of Total Selenium in Biofortified Allium sp. following Piazselenol Formation and Micro-Solid-Phase Extraction
Source: Molecules. 2021 Nov 6;26(21):6730. doi: 10.3390/molecules26216730 (PMC8588065; doi:10.3390/molecules26216730)
Supplement: Supplementary file 1 [file molecules-26-06730-s001.zip › molecules-1427396-supplementary.pdf]

CRM	Element	Reference value+uncertainty	Concentration
CSM-3Mushroom Powder	As	0.651±0.026	
	Cd	1.229±0.110	
	Cr	5.79±0.80	
	Cu	18.73±0.70	mg/kg
	Hg	2.849±0.104	
	Pb	1.863±0.108	
	Se	17.43±1.36	
	Zn	113.30±3.28	
SRM 3280 Multivitamin	B	0.141±0.007	
	Ca	110.7±5.3	
	Cl^-	53.0±2.3	
	Cu	1.40±0.17	
	I	0.1327±0.0066	
	Fe	12.35±0.91	
	Mg	67.8±4.0	
	Mn	1.44±0.11	mg/g
	P	75.7±3.2	
	K	53.1±7.0	
	Zn	10.15±0.81	
	As	0.132±0.044	
	Cr	93.7±2.7	
	Pb	0.2727±0.0024	
	Mo	70.7±4.5	
	Ni	8.43±0.30	
	Se	17.42±0.45	
	Cd	80.15±0.86	
CRM 025050 Metals in soil	Al	7.637±0.737	
	Sb	<3.2	
	As	339±20	
	Ba	1.839±0.428	
	Be	0.33±0.06	
	B	17.2	
	Cd	369±19	
	Ca	28.320±1.901	
	Cr	441±22	
	Co	4.07±0.51	
	Cu	7.76±0.73	
	Fe	9.439±0.516	
	Pb	1.447±0.088	mg/kg
	Mg	4.376±0.208	
	Mn	173±6	
	Hg	99.8±18.0	
	Mo	<0.8±	
	Ni	12.2±1.4	
	K	1.992±0.199	
	Se	518±31	
	Si	171±65	
	Ag	132±136	
	Na	313±26	
	Sr	408	

Tl	<4.8
V	19.3±2.0
Zn	51.8±3.4

TableS1. Certified mass values for selected elements in CSM-3Mushroom Powder, SRM 3280 Multivitamin and CRM 025050 Metals in soil.
